# The predictors of death within 1 year in acute ischemic stroke patients based on machine learning

**DOI:** 10.3389/fneur.2023.1092534

**Published:** 2023-02-23

**Authors:** Kai Wang, Longyuan Gu, Wencai Liu, Chan Xu, Chengliang Yin, Haiyan Liu, Liangqun Rong, Wenle Li, Xiu'e Wei

**Affiliations:** ^1^Department of Neurology, The Second Affiliated Hospital of Xuzhou Medical University, Xuzhou, Jiangsu, China; ^2^Key Laboratory of Neurological Diseases, The Second Affiliated Hospital of Xuzhou Medical University, Xuzhou, Jiangsu, China; ^3^Department of Neurosurgery, The Affiliated Hospital of Xuzhou Medical University, Xuzhou, Jiangsu, China; ^4^Department of Orthopaedic Surgery, The First Affiliated Hospital of Nanchang University, Nanchang, China; ^5^Department of Dermatology, Xianyang Central Hospital, Xianyang, China; ^6^Faculty of Medicine, Macau University of Science and Technology, Taipa, Macao SAR, China; ^7^The State Key Laboratory of Molecular Vaccinology and Molecular Diagnostics and Center for Molecular Imaging and Translational Medicine, School of Public Health, Xiamen University, Xiamen, China

**Keywords:** ischemic stroke, biomarkers, machine learning, prediction model, web calculator

## Abstract

**Objective:**

To explore the predictors of death in acute ischemic stroke (AIS) patients within 1 year based on machine learning (ML) algorithms.

**Methods:**

This study retrospectively analyzed the clinical data of patients hospitalized and diagnosed with AIS in the Second Affiliated Hospital of Xuzhou Medical University between August 2017 and July 2019. The patients were randomly divided into training and validation sets at a ratio of 7:3, and the clinical characteristic variables of the patients were screened using univariate and multivariate logistics regression. Six ML algorithms, including logistic regression (LR), gradient boosting machine (GBM), extreme gradient boosting (XGB), random forest (RF), decision tree (DT), and naive Bayes classifier (NBC), were applied to develop models to predict death in AIS patients within 1 year. During training, a 10-fold cross-validation approach was used to validate the training set internally, and the models were interpreted using important ranking and the SHapley Additive exPlanations (SHAP) principle. The validation set was used to externally validate the models. Ultimately, the highest-performing model was selected to build a web-based calculator.

**Results:**

Multivariate logistic regression analysis revealed that C-reactive protein (CRP), homocysteine (HCY) levels, stroke severity (SS), and the number of stroke lesions (NOS) were independent risk factors for death within 1 year in patients with AIS. The area under the curve value of the XGB model was 0.846, which was the highest among the six ML algorithms. Therefore, we built an ML network calculator (https://mlmedicine-de-stroke-de-stroke-m5pijk.streamlitapp.com/) based on XGB to predict death in AIS patients within 1 year.

**Conclusions:**

The network calculator based on the XGB model developed in this study can help clinicians make more personalized and rational clinical decisions.

## 1. Introduction

Acute ischemic stroke (AIS) is a disease caused by the occlusion of cerebral arteries, accompanied by brain tissue infarction and neuronal cell damage, causing severe trauma to the body. AIS is the leading cause of disability in adults and the primary cause of human death worldwide (1, 2). In 2019, there were 7,630,800 cases of AIS globally, an 87.55% increase compared to the previous 30 years. The high morbidity, mortality, and disability rates associated with AIS impose a severe economic burden on society and families (3). Several factors may have a significant impact on the pathogenesis and prognosis of patients with AIS, including the immune inflammatory response during AIS development, with the involvement of different pathways and sources of activated inflammatory factors, and is an important regulator of stroke progression, post-stroke damage, cerebral function repair and death (4–6). Approximately 10% of AIS patients, representing a type of morbidity, experience a fatal event within 1 year (7). There is an urgent need to identify the early and effective predictors of death 1 year after the onset of AIS. The construction of a model of death prediction in stroke patients within 1 year could provide clinicians with a reliable tool to assess the condition of their patients. However, there are few reports in this area.

ML-assisted clinical decision-making and analysis have been widely used in clinical settings (8–11), especially in the screening phase of big data feature variables (12, 13). The superior performance demonstrated by ML algorithms in medical big data makes it possible to obtain better predictive tools than traditional statistical models under certain conditions. However, few studies have been conducted to screen the risk factors of death in AIS patients within 1 year using ML algorithms.

Therefore, this study aimed to develop and validate an interpretable ML model that used clinically relevant variables to predict death within 1 year in AIS patients and construct an easy-to-use web calculator as a convenient and practical protective tool for clinical practitioners to provide valid information for AIS patients.

## 2. Materials and methods

### 2.1. Subjects

Patients who were hospitalized in the Department of Neurology of the Second Affiliated Hospital of Xuzhou Medical University and diagnosed with AIS between August 2017 and July 2019 were retrospectively analyzed. A total of 677 patients with AIS were included in this study, 32 of whom died after admission and during follow-up. The study was approved by the Ethics Committee of the Second Affiliated Hospital of Xuzhou Medical University [ethics number: [2020] 081603], and all patients provided written informed consent.

### 2.2. Inclusion and exclusion criteria

The inclusion criteria were a diagnosis of AIS in accordance with the World Health Organization criteria, and the time between onset and hospital admission did not exceed 24 h. The exclusion criteria were: (1) incomplete clinical data, (2) those with severely abnormal organ function, (3) inadequate ancillary investigations, (4) follow-up of <1 year, and (5) Patients who discontinued treatment for various reasons according to their relatives.

### 2.3. Methods

#### 2.3.1. Observational variables

In this study, clinical data were collected from the enrolled patients, including demographics (age and sex); vascular risk factors (hypertension, diabetes mellitus, and ischemic heart disease); baseline blood pressure [systolic blood pressure (SBP) and diastolic blood pressure (DBP)]; Trial of Org 10 172 in Acute Stroke Treatment (TOAST) [large-artery atherosclerosis, cardioembolism, small-vessel occlusion, acute stroke of other determined etiology, stroke of undetermined etiology]; stroke severity (SS) [defined as mild stroke according to the National Institutes of Health Stroke Scale (NIHSS) scores of ≤ 8, moderate-to-severe stroke according to NIHSS scores of ≥9; all assessments completed on admission]; magnetic resonance imaging (MRI) features [stroke distribution (SD; anterior circulation, posterior circulation, and anterior/posterior circulation), side of hemisphere (SOH; left, right, and bilateral), number of stroke lesions (NOSs; single and multiple stroke lesions), site of stroke lesions (SOSs; cortical, cortico-subcortical, subcortical, brainstem, and cerebellum)]; laboratory tests [total cholesterol, triglycerides, low-density lipoprotein (LDL), fasting blood glucose (FBG), homocysteine (HCY), uric acid (UA), fibrinogen (FIB), myoglobin (MB), C-reactive protein (CRP), D-dimer brain natriuretic peptide (BNP), HBALC, neuron-specific enolase (NSE), and S-100β levels], treatment regimen [intravenous thrombolysis, arterial thrombolysis, antiplatelet, anticoagulation, statin, and proton pump inhibitor therapy (PPI)]; and stroke comorbidities [dysphagia and stroke-associated pneumonia (SAP)].

#### 2.3.2. Statistical methods

This study used R version 4.0.5 software for data processing and statistical analyses. Continuous variables are expressed as the median or interquartile range (IQR) while categorical variables are presented as frequencies (percentage, %). The continuous variables were compared by independent samples *t*-tests and the categorical variables were compared using χ^2^-tests. Understanding the relationship between the independent and dependent variables was clinically meaningful and *P*-values of <0.05 were considered statistically significant (two-sided).

#### 2.3.3. Modeling of machine learning algorithms

Univariate and multivariate logistic regression analyses were used to assess the risk factors of death within 1 year in the training group study population. The odds ratio (OR) and 95% confidence interval (CI) were calculated, with an OR of > 1 indicating that the variable was a risk factor, and *P* < 0.05 considered to indicate a statistically significant difference. Then, the factors that were significant in both univariate and multivariate logistic regression were included and subjected to stepwise regression analysis. The factors selected by stepwise regression were used as input variables to construct ML models.

The ML algorithm process was based on Python (V3.7) software and the scikit-learn (version 0.24) library. First, the original dataset was randomly divided into training and test sets at a ratio of 7:3. Then, six machine algorithms [logistic regression (LR), gradient boosting machine (GBM), extreme gradient boosting (XGB), random forest (RF), decision tree (DT), and naive Bayes classifier (NBC)] were used to analyze the data and construct the model. To validate the predictive power of the model, the 10-fold cross-validation method was used for internal validation against the training group. The random search method was used to adjust the hyperparameters of the models.

In the test group, the area under the receiver operating characteristic curve (ROC-AUC), classification accuracy, recall, specificity, and F1 score were used to evaluate the prediction models. We also plotted the prediction recall curve (PRC) as a complementary metric to evaluate the model performance.

#### 2.3.4. Interpretation of the model and importance of features

To illustrate the risk factors of death within 1 year in AIS patients, Shapley Additive explanation (SHAP) analysis was used to interpret the predictive models ranked in terms of feature importance. SHAP analysis is a tool proposed by Lloyd Shapley in game theory to explain the output of machine learning models. The core idea is to calculate the marginal contribution of a variable feature when it is added to the model, and then to interpret the global and local levels of the “black box model” in an additive explanatory model (14, 15). That is, it can assign predictive values to each feature and evaluate and visualize the contribution of each feature to the outcome of the machine learning model (16). Ultimately, a web-based calculator based on the best-performing model was created for inputting patient data to facilitate the clinicians' assessment of death within 1 year in AIS patients.

## 3. Results

### 3.1. Baseline patient data characteristics

In this study, clinical information was collected on 677 AIS patients, of whom 645 survived and 32 died of AIS (Table 1). In the observed population, 383 patients (56.6%) were aged <60 years and 294 patients (43.4%) were aged ≥ 60 years, 398 (58.8%) were male and 279 (41.2%) were female. AIS lesions occurred in 270 (39.9%) patients in the anterior circulation, 252 (37.2%) in the posterior circulation, and 155 (22.9%) in both anterior and posterior circulations. The distribution of lesions in the left and right hemispheres was approximately equal, with 283 (41.8%) in the left and 270 (39.9%) in the right, and a relatively small number [124 (18.3%)] in the bilateral cerebral hemispheres. The location of the lesions was mainly subcortical in 186 patients (27.5%), cortical and cortico-subcortical in 155 patients (22.9%), the brainstem in 104 patients (15.4%), and the cerebellum in 77 patients (11.4%). Four hundred and seventy patients (69.4%) had a single AIS lesion, while multiple lesions were found in only 207 patients (30.6%). Two hundred and four patients (30.1%) received intravenous thrombolytic therapy, and 473 (69.9%) did not. Six hundred and forty-four patients (95.1%) did not receive arterial thrombolytic therapy, and 33 (4.9%) did. Five hundred and fifty-five patients (82.0%) received antiplatelet therapy, and 122 (18.0%) did not. Five hundred and seventy-six patients (85.1%) did not receive anticoagulation therapy, and 101 (14.9%) did. The majority of the patients (574, 84.8%) received statin therapy and 103 (15.2%) did not.

**Table 1:** Baseline table of whether stroke patients died within 1 year.

**Characteristics**		**Overall (*N* = 677)**	**No (*N* = 645)**	**Yes (*N* = 32)**	***P*-value**
Age, *n* (%)	≤ 60	383 (56.6)	362 (56.1)	21 (65.6)	0.381
	>60	294 (43.4)	283 (43.9)	11 (34.4)	
Gender, *n* (%)	Female	279 (41.2)	263 (40.8)	16 (50.0)	0.395
	Male	398 (58.8)	382 (59.2)	16 (50.0)	
SD, *n* (%)	Anterior circulation	270 (39.9)	258 (40.0)	12 (37.5)	0.082
	Posterior circulation	252 (37.2)	235 (36.4)	17 (53.1)	
	Anterior/posterior circulation	155 (22.9)	152 (23.6)	3 (9.4)	
SOH, *n* (%)	Left	283 (41.8)	271 (42.0)	12 (37.5)	0.87
	Right	270 (39.9)	256 (39.7)	14 (43.8)	
	Bilateral	124 (18.3)	118 (18.3)	6 (18.8)	
SOS, *n* (%)	Cortex	155 (22.9)	149 (23.1)	6 (18.8)	0.95
	Cortex-subcortex	155 (22.9)	147 (22.8)	8 (25.0)	
	Subcortex	186 (27.5)	176 (27.3)	10 (31.2)	
	Brainstem	104 (15.4)	100 (15.5)	4 (12.5)	
	Cerebellum	77 (11.4)	73 (11.3)	4 (12.5)	
NOS, *n* (%)	Single stroke lesion	470 (69.4)	453 (70.2)	17 (53.1)	0.064
	Multiple stroke lesions	207 (30.6)	192 (29.8)	15 (46.9)	
Thrombolysis, *n* (%)	No	473 (69.9)	448 (69.5)	25 (78.1)	0.398
	Yes	204 (30.1)	197 (30.5)	7 (21.9)	
Thrombectomy, *n* (%)	No	644 (95.1)	614 (95.2)	30 (93.8)	0.665
	Yes	33 (4.9)	31 (4.8)	2 (6.2)	
Antiplatelet, *n* (%)	No	122 (18.0)	117 (18.1)	5 (15.6)	0.9
	Yes	555 (82.0)	528 (81.9)	27 (84.4)	
Anticoagulation, *n* (%)	No	576 (85.1)	553 (85.7)	23 (71.9)	0.041
	Yes	101 (14.9)	92 (14.3)	9 (28.1)	
Statin, *n* (%)	No	103 (15.2)	98 (15.2)	5 (15.6)	1
	Yes	574 (84.8)	547 (84.8)	27 (84.4)	
PPI, *n* (%)	No	535 (79.0)	519 (80.5)	16 (50.0)	<0.001
	Yes	142 (21.0)	126 (19.5)	16 (50.0)	
SS, *n* (%)	No	385 (56.9)	380 (58.9)	5 (15.6)	<0.001
	Yes	292 (43.1)	265 (41.1)	27 (84.4)	
SAP, *n* (%)	No	512 (75.6)	494 (76.6)	18 (56.2)	0.016
	Yes	165 (24.4)	151 (23.4)	14 (43.8)	
SBP, median [Q1, Q3]		143.0 [132.0, 156.0]	143.0 [132.0, 156.0]	144.0 [134.8, 156.2]	0.678
DBP, median [Q1, Q3]		87.0 [74.0, 97.0]	87.0 [74.0, 97.0]	87.0 [74.0, 98.2]	0.492
Cholesterol, median [Q1, Q3]		5.3 [4.4, 6.2]	5.3 [4.4, 6.2]	5.4 [4.7, 6.0]	0.815
Triglyceride, median [Q1, Q3]		2.2 [1.9, 2.4]	2.2 [1.9, 2.4]	2.1 [1.9, 2.4]	0.801
LDL, median [Q1, Q3]		4.8 [4.3, 4.9]	4.8 [4.3, 4.9]	4.7 [4.5, 4.8]	0.69
FBG, median [Q1, Q3]		5.3 [4.6, 5.8]	5.2 [4.6, 5.8]	5.7 [5.2, 6.2]	0.003
HBALC, median [Q1, Q3]		5.6 [5.3, 5.9]	5.6 [5.3, 5.9]	5.8 [5.5, 6.1]	0.023
HCY, median [Q1, Q3]		15.8 [12.7, 19.4]	15.5 [12.6, 19.1]	20.4 [17.8, 22.7]	<0.001
UA, median [Q1, Q3]		349.8 [309.8, 408.1]	350.1 [310.8, 407.6]	335.5 [290.7, 435.4]	0.573
MB, median [Q1, Q3]		97.7 [75.1, 147.8]	97.0 [74.9, 144.7]	106.6 [78.9, 236.5]	0.078
CRP, median [Q1, Q3]		12.6 [7.7, 17.6]	11.9 [7.5, 17.1]	20.5 [17.3, 25.0]	<0.001
FIB, median [Q1, Q3]		4.3 [4.0, 4.8]	4.4 [4.0, 4.8]	4.2 [3.8, 4.6]	0.134
D-dimer, median [Q1, Q3]		174.0 [133.0, 221.0]	174.0 [133.0, 221.0]	171.5 [132.0, 216.5]	0.844
BNP, median [Q1, Q3]		93.0 [73.0, 162.0]	93.0 [73.0, 162.0]	121.5 [77.0, 177.2]	0.25
NSE, median [Q1, Q3]		16.2 [12.7, 18.6]	16.2 [12.7, 18.6]	17.6 [12.5, 19.4]	0.197
S100β, median [Q1, Q3]		275.0 [224.0, 290.0]	275.0 [223.0, 289.0]	278.0 [248.2, 311.2]	0.111

Overall: All patients, No, Patients who did not die, Yes, Patients who died.

SD, stroke distribution; SOH, side of hemisphere; NOS, number of stroke lesions; SOS, site of stroke lesions; LDL, low-density lipoprotein; FBG, fasting blood glucose; HCY, homocysteine; UA, uric acid; FIB, fibrinogen; MB, myoglobin; CRP, C-reactive protein; BNP, brain natriuretic peptide; NSE, neuron-specific enolase; PPI, proton pump inhibitor therapy; SAP, stroke-associated pneumonia.

The median systolic and diastolic blood pressure was 143 mmHg (IQR 132.0,156.0) and 87 mmHg (IQR 74.0, 97.0), respectively. Total cholesterol, triglycerides, HDL, blood HCY, blood UA, and median FIB, MB, ultrasensitive CRP, D-dimer BNP, atrial natriuretic peptide, NSE, and S-100β were 5.3 mmol/L [4.4, 6.2], 2.2 mmol/L [1.9, 2.4], 4.8 mmol/L [4.3, 4.9], 15.7 μmol/L [12.4, 19.1], 349.8 μmol/L [309.8, 408.1], 4. 3 g/L [4.0, 4.8], 97. 7 ng/mL [75.1, 147.8], 12. 2 mg/L [7.2, 18.1], 174.0 ng/mL [133.0, 221.0], 93.0 ng/mL [73.0, 162.0], 16.2 ng/mL [12.7, 18.6], and 275.0 ng/mL [224.0, 290.0], respectively.

### 3.2. Univariate and multivariate regression analysis of death within 1 year in AIS patients

In the univariate regression analysis of death within 1 year in AIS patients (Table 2), there was a statistically significant difference (*P* < 0.05) in the overall population for death within 1 year according to NOS, FBG, HBALC, MB, and CRP levels, as well as anticoagulation therapy, PPI treatment, and SS.

**Table 2:** Univariate and multivariate logistic regression analysis of 1-year death in AIS patients.

**Characteristics**	**Univariate logistic analysis**			**Multivariate logistic analysis**		
	**OR**	**95% CI**	***P*-value**	**OR**	**95% CI**	***P*-value**
Age:						
≤ 60	Ref.	Ref.	Ref.			
>60	0.67	(0.31–1.40)	0.297			
Gender:						
Female		Ref.	Ref.			
Male	0.69	(0.33–1.42)	0.309			
SD:						
Anterior circulation	Ref.	Ref.	Ref.			
Posterior circulation	1.55	(0.72–3.41)	0.26			
Anterior/posterir circulation	0.44	(0.09–1.44)	0.186			
SOH:						
Left		Ref.	Ref.			
Right	1.23	(0.55–2.78)	0.607			
Bilateral	1.16	(0.39–3.11)	0.774			
SOS:						
Cortex		Ref.	Ref.			
Cortex-subcortex	1.34	(0.45–4.26)	0.599			
Subcortex	1.4	(0.50–4.27)	0.53			
Brainstem	1.01	(0.24–3.72)	0.993			
Cerebellum	1.38	(0.33–5.11)	0.642			
NOS:						
Single stroke lesion	Ref.	Ref.	Ref.	Ref.	Ref.	Ref.
Multiple stroke lesions	2.08	(1.00–4.28)	0.049	3.44	(1.41–8.36)	0.007
Thrombolysis:						
No	Ref.	Ref.	Ref.			
Yes	0.65	(0.25–1.45)	0.305			
Thrombectomy:						
No	Ref.	Ref.	Ref.			
Yes	1.41	(0.20–5.00)	0.671			
Antiplatelet:						
No	Ref.	Ref.	Ref.			
Yes	1.17	(0.47–3.56)	0.755			
Anticoagulation:						
No	Ref.	Ref.	Ref.	Ref.	Ref.	Ref.
Yes	2.37	(1.00–5.16)	0.049	0.97	(0.35–2.67)	0.951
Statin:						
No		Ref.	Ref.			
Yes	0.94	(0.38–2.89)	0.91			
PPI:						
No	Ref.	Ref.	Ref.	Ref.	Ref.	Ref.
Yes	4.11	(1.98–8.54)	<0.001	1.65	(0.62–4.38)	0.317
SS:						
No	Ref.	Ref.	Ref.	Ref.	Ref.	Ref.
Yes	7.53	(3.09–22.8)	<0.001	3.12	(1.03–9.83)	0.046
SAP:						
No	Ref.	Ref.	Ref.	Ref.	Ref.	Ref.
Yes	2.55	(1.21–5.25)	0.015	0.98	(0.36–2.68)	0.971
SBP	1.01	(0.99–1.03)	0.242			
DBP	1.01	(0.99–1.04)	0.303			
Cholesterol	1	(0.75–1.32)	0.988			
Triglyceride	0.77	(0.27–2.16)	0.616			
LDL	1.08	(0.66–1.77)	0.769			
FBG	1.53	(1.09–2.14)	0.015	2.15	(0.89–5.21)	0.088
HBALC	2.34	(1.01–5.43)	0.048	0.56	(0.07–4.54)	0.59
HCY	1.32	(1.20–1.45)	<0.001	1.29	(1.16–1.45)	<0.001
UA	1	(0.99–1.00)	0.732			
MB	1.01	(1.00–1.01)	0.003	1	(0.99–1)	0.746
CRP	1.17	(1.11–1.22)	<0.001	1.15	(1.07–1.23)	<0.001
FIB	0.62	(0.35–1.10)	0.106			
D-dimer	1	(0.99–1.01)	0.761			
BNP	1	(1.00–1.01)	0.157			
NSE	1.06	(0.96–1.17)	0.214			
S100β	1.01	(1.00–1.02)	0.082			

All parameters that were statistically different in the univariate analysis above were included in the multivariate logistic regression analysis. The results suggested that NOS (OR = 3.44, 95% CI: 1.41 – 8.36, *P* = 0.007), HCY (OR = 1.29, 95% CI: 1.16 – 1.45, *P* < 0.001), CRP (OR = 1.15, 95% CI: 1.07 – 1.23, *P* < 0.001), and SS (OR = 3.12, 95% CI: 1.03 – 9.83, *P* = 0.046) were independent predictors of death within 1 year in AIS patients.

### 3.3. Machine learning model building and validation

**Figure 1:**
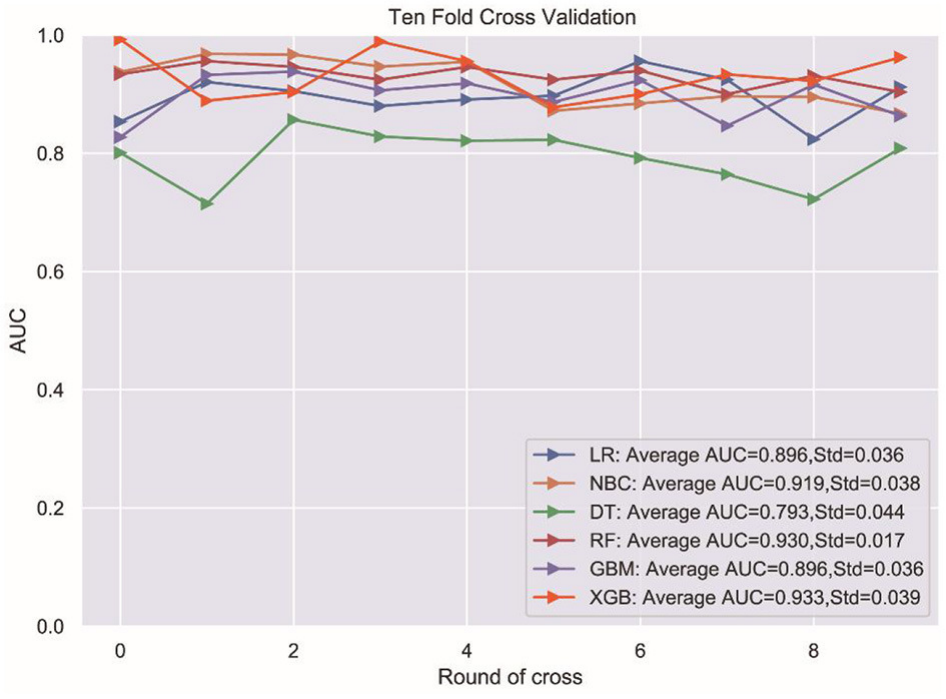
Ten-fold cross validation test. LR, logistic regression; NBC, Naive Bayesian classification; DT, Decision Tree; RF, Random Forest; GBM, gradient boosting machine; XGB, extreme gradient boosting.

**Figure 2:**
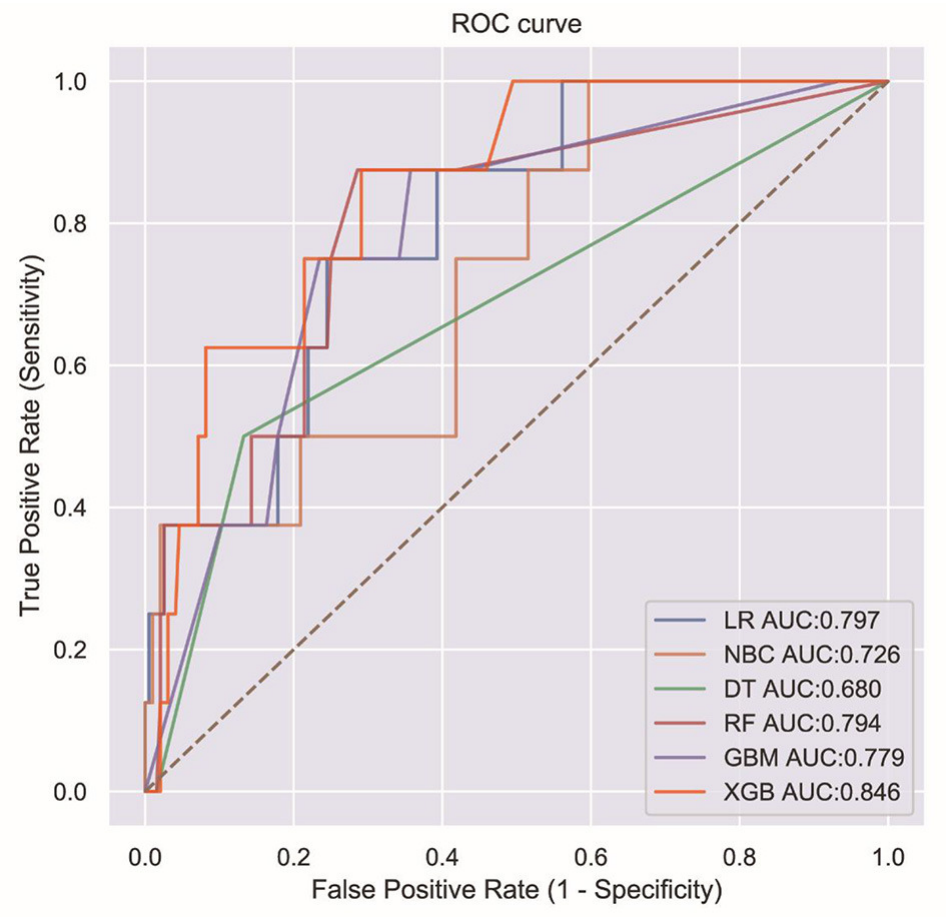
ROC curves for six ML algorithms.

**Figure 3:**
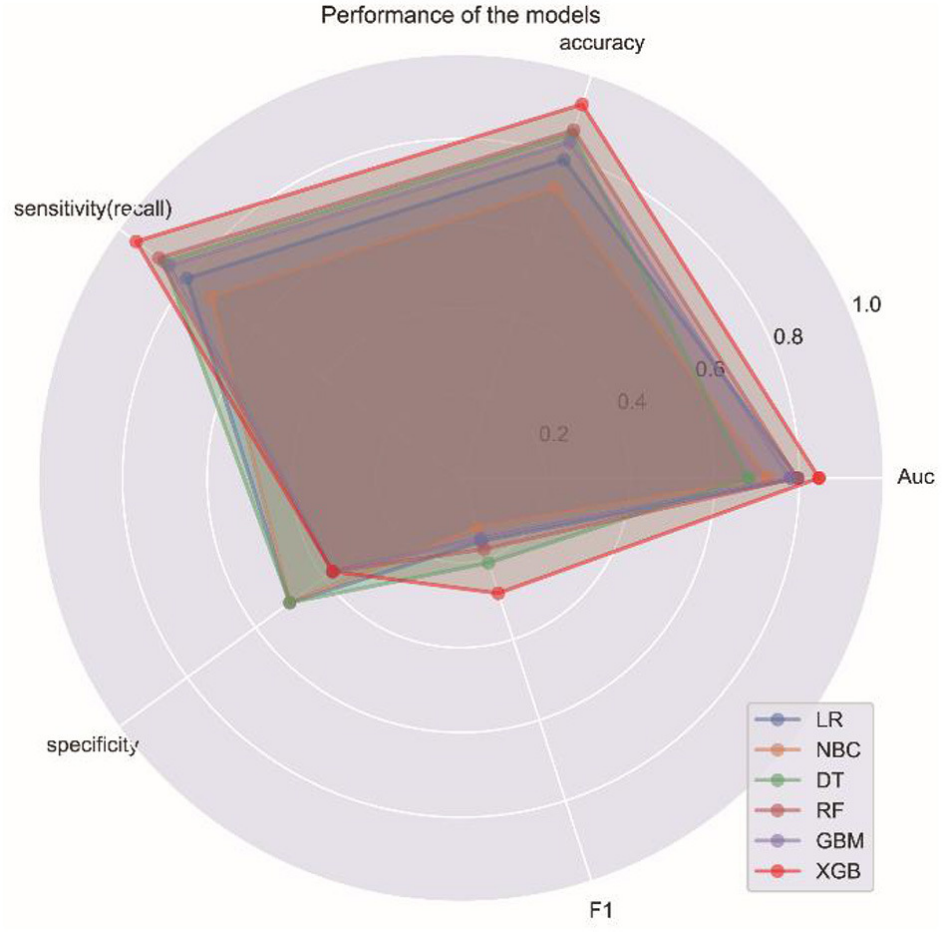
Radar graph showing the comprehensive prediction performance of six ML algorithms.

To compare the predictive performance of the six ML algorithm models, this study performed 10-fold cross-validation within the training group. The results are shown in Figure 1. Figure 2 shows the ROC curves of the predictive performance differences of the six ML algorithm models after external validation, and Figure 3 shows the result of radar plot analysis, which is a blanket, clear, intuitive, and easy-to-judge analysis and is suitable for comprehensive evaluation as it can show the AUC value, accuracy, recall, and F1 value of the models in multiple dimensions (Figure 3, Table 3) to more clearly reflect the performance of the models. The PRC curves of the mortality prediction model are shown in Supplementary Figure 1.

**Table 3:** The result of specific performance of six ML algorithm models.

**Scoring**	**LR**	**NBC**	**DT**	**RF**	**GBM**	**XGB**
Auc	0.797	0.726	0.680	0.794	0.779	0.846
Accuracy	0.789	0.721	0.853	0.863	0.833	0.926
Sensitivity (recall)	0.801	0.730	0.867	0.883	0.852	0.949
Specificity	0.500	0.500	0.500	0.375	0.375	0.375
F1	0.157	0.123	0.211	0.176	0.150	0.286

The results suggest that the XGB model performed best in predicting death within 1 year in AIS patients after a comprehensive evaluation. The remaining models were ranked in descending order according to their predictive performance.

In summary, we finally adopted the XGB model as the preferred predictive model.

### 3.4. Relative importance of variables in ML algorithms

**Figure 4:**
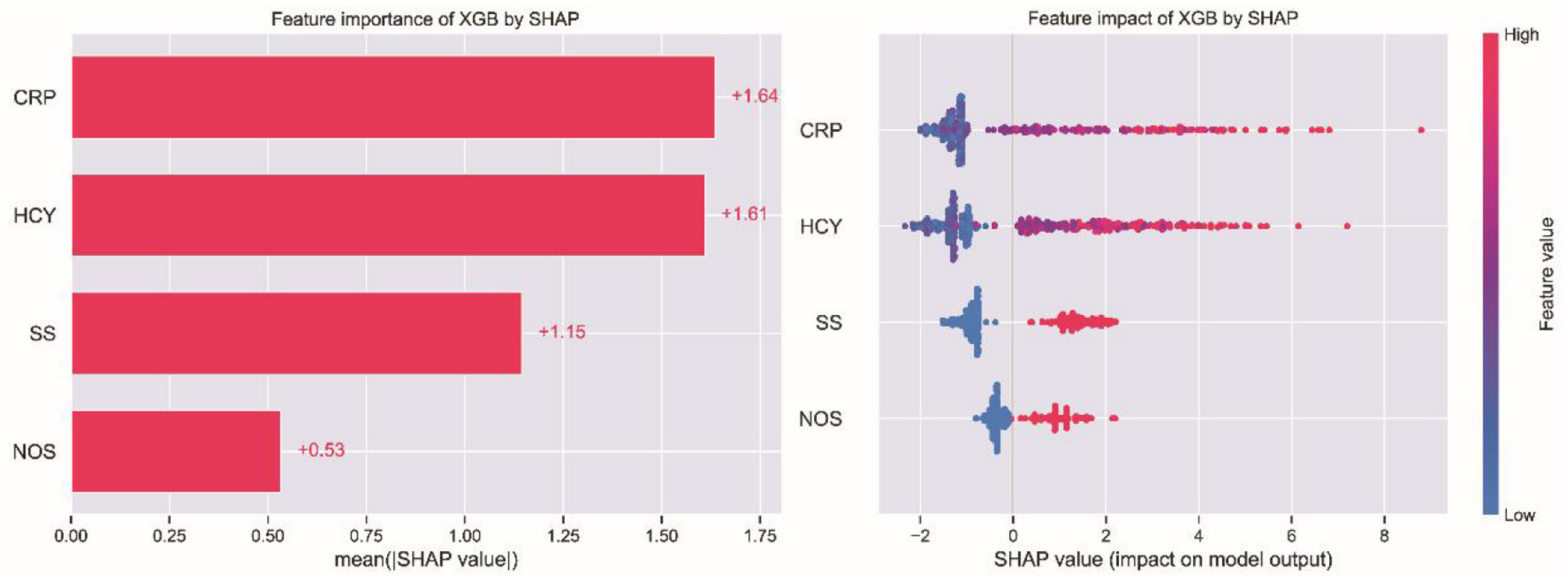
Patient clinical feature importance of XGB.

A SHAP interpretability study was used to analyze the results of the ML models. Generally, the higher the SHAP value of a feature, the higher the probability of the occurrence of the target event. In SHAP analysis, red represents the eigenvalues with positive impact on the model and blue represents the eigenvalues with negative impact on the model (17). The results of the study suggest that SS was the most important variable, followed by CRP, HCY, and NOS in descending order of importance, as shown in Figure 4.

### 3.5. The web calculator

**Figure 5:**
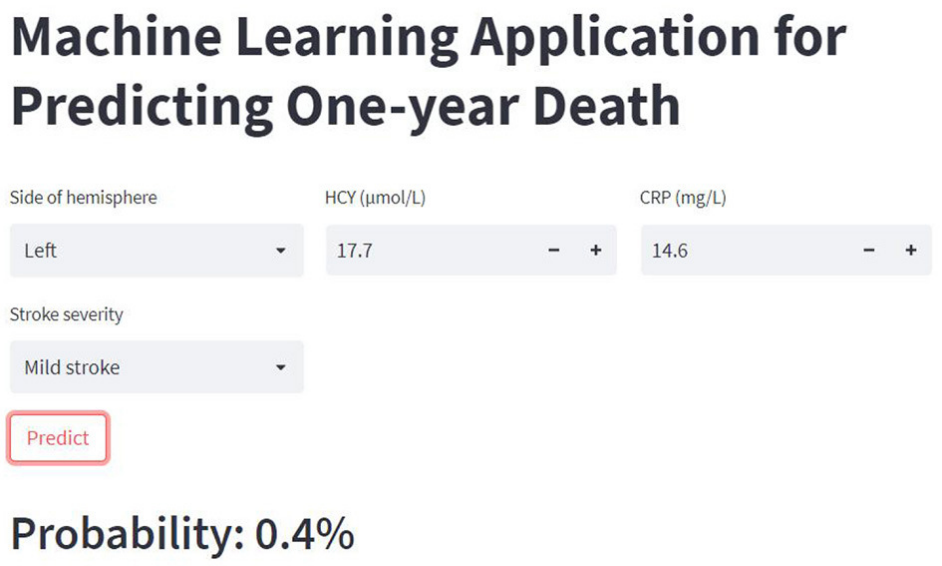
The web-based calculator for predicting 1-year death in AIS patients.

A web-based calculator based on the XGB model was developed in this study. By entering the clinical characteristic variables of a patient with AIS, clinicians could predict their risk of death within 1 year (https://mlmedicine-de-stroke-de-stroke-m5pijk.streamlitapp.com/; Figure 5).

## 4. Discussion

In this study, we retrospectively analyzed the clinical data of AIS patients and developed a web-based calculator with ML algorithms to predict the risk of death within 1 year. The accuracy and rationality of the model were validated by 10-fold cross-validation, allowing the model to be used for clinical practice to help clinicians make more rational treatment decisions.

ML is an emerging field of medicine that has demonstrated an extraordinary ability to handle large, complicated, and disparate data, and is the future of biomedical research, personalized medicine, and computer-aided diagnosis. It holds the promise of significantly advancing global healthcare (18, 19). Unlike traditional predictive models, ML is very good at discovering complex structures in selected variables in high-dimensional data and can easily combine a large number of variables (20, 21). ML has been reported to improve the predictive accuracy of long-term prognoses for AIS patients (8, 10).

In this study, six ML methods were used to analyze and construct a model of death prediction within 1 year in AIS patients, and the performance of the six ML algorithms was compared to each other. The XGB algorithm performed best (Figure 1), with a better AUC value than the other five algorithmic models, and the highest accuracy, sensitivity, and F1 score. Therefore, the XGB algorithm model was finally chosen.

ML models are often considered to be a black box where is difficult to explain the predictive performance, and it becomes extremely important to study the interpretability of machine learning models. Therefore, this study attempted to introduce SHAP analysis, a new method for interpreting various black-box ML models that have been previously validated based on their interpretability performance. It can achieve both local and global interpretability and has a solid theoretical foundation compared to other methods (22). The SHAP analysis used in this study could interpret the model prediction results well, and its intuitive visualization is more easily accepted. This study further built a web-based calculator to estimate the probability of death within 1 year in AIS patients to make better use of the model.

AIS is characterized by a high morbidity rate, which increases the economic burden on society and families (23). It is significant to explore the factors influencing the risk of death within 1 year for patients. In this study, the mortality rate of AIS patients within 1 year was only 4.7% (32/677), which was significantly lower than the 10% reported in previous studies (7), probably because of the exclusion of those whose families discontinued treatment for various reasons. Previously, an 8-point scoring system was constructed to predict the risk of death within 7 days of hospitalization (24). Factors influencing death within 6 months of stroke onset were also reported, with variables such as the Barthel index and platelet/lymphocyte ratio screened by LASSO regression and multiple logistic regression (25). A 30-year stroke burden predictive model was established (26). In contrast, unlike many previous studies, this study innovatively used machine learning algorithms to screen variables and, to our knowledge, was the first to develop a predictive model using machine learning algorithms to assess the probability of death within 1 year in patients with AIS.

There is a growing body of research on the relationship between serum inflammatory biomarkers and AIS. A number of studies showed that AIS could induce an inflammatory response, which plays a major role in late ischemic damage to the brain parenchyma, and that inflammatory responses caused by various clinical factors could lead to an increase in inflammatory factors (27, 28). It is also an inflammatory factor that can indirectly indicate the presence of pathogenic microorganisms in patients when it is upraised, which can help the physician in the diagnosis and treatment. In this study, we concluded that CRP levels were the most important predictor of death within 1 year in AIS patients. Elevated CRP levels were previously reported to reflect the severity of AIS, correlate with stroke subtype and risk stratification (27, 28), and be an independent predictor of long-term mortality after ischemic stroke (29). Elevated CRP levels can lead to increased mortality after stroke, which may be related to inflammation-induced endothelial cell dysfunction and platelet activation (30). HCY is a sulfur-containing non-essential amino acid produced by metabolism *in vivo* as a derivative of methionine cycle demethylation. It is also an inflammatory substance that induces the activation of nuclear factor (NF)-kB, which is a transcription factor common to inflammation and the immune response. Elevated levels of HCY are associated with a variety of diseases, which may lead to endothelial dysfunction, neurotoxicity, and the upregulation of thrombogenic factors. At the same time, monitoring HCY levels may provide a good indication of the development of related diseases (31). Previous studies also showed that elevated HCY levels were associated with AIS dysfunction and recurrent stroke (32). A multicenter study suggested that high levels of serum HCY were an independent predictor of early neurological deterioration in AIS patients (33). This study concluded that HCY levels significantly influenced the risk of death within 1 year in AIS patients. The risk of death in patients with high HCY having 1.29 times (95% CI 1.16 – 1.45) compared to ones with normal HCY.

The NIHSS is a common scale used in neurology as a quantitative indicator of disease severity (34). The present study classified SS with the help of the NIHSS scale, and an NIHSS score of ≥9 was defined as moderate-to-severe stroke. Fischer et al. (35) suggested that patients with low NIHSS scores tended to have a better prognosis, which is consistent with the current study. The present study concluded that SS had a significant influence on death within 1 year in AIS patients. The risk of death in patients with moderate to severe stroke having 3.12 times (95% CI 1.03 – 9.83) higher than those with mild stroke.

Neurological deficits have been associated with lesions in different brain regions (36, 37), but the relationship between the number of lesions and AIS has rarely been reported. In this study, the number of lesions was innovatively included in the analysis, and the results suggested that the number of lesions was a significant factor in death within 1 year in AIS patients. The risk of death in patients with multiple lesions was 3.44 times (95% CI: 1.41 – 8.36) higher than patients with a single lesion.

There were several limitations to this study. First, the retrospective study design may have introduced selection bias, while the data imbalance that emerged from real-world studies resulted in PRC effects without the AUC number. Secondly, although our model showed good performance, its data source was limited to one medical center, which may limit its generalizability, and we will follow up with an additional multicenter study. Thirdly, further independent external validation is needed to confirm these findings. Finally, we collected AIS-related variables as comprehensively as possible, but there were still some important variables that were not available in a timely manner, which may also limit the generalizability of the study. Future research is needed to examine this issue further.

## 5. Conclusion

The results of this study suggest that serum inflammatory markers (CRP and HCY), SS, and NOS are independent risk factors of death within 1 year in AIS patients. The XGB algorithm showed good performance as a tool to predict death within 1 year in AIS patients. Using this web-based calculator can effectively prevent death, reduce mortality, and assist physicians in making treatment decisions.

## Data availability statement

The raw data supporting the conclusions of this article will be made available by the authors, without undue reservation.

## Ethics statement

The study was written approved by the Ethics Committee of the Second Affiliated Hospital of Xuzhou Medical University [ethics number: [2020] 081603]. The patients/participants provided their written informed consent to participate in this study.

## Author contributions

WLi, LR, and XW completed the study design. KW, WLiu, and WLi performed the study and collected and analyzed the data. LG and WLi drafted the manuscript. LR, XW, KW, and HL provided the expert consultations and suggestions. CX and CY conceived of the study, participated in its design and coordination, and helped to embellish language. All authors reviewed the final version of the manuscript.

## Supplementary material

Supplementary Figure 1 Precision recall curve (PRC) for machine learning algorithm.
