# Supplementary figures and images for: The predictors of death within 1 year in acute ischemic stroke patients based on machine learning
Source: Front Neurol. 2023 Feb 23;14:1092534. doi: 10.3389/fneur.2023.1092534 (PMC9998042; doi:10.3389/fneur.2023.1092534)

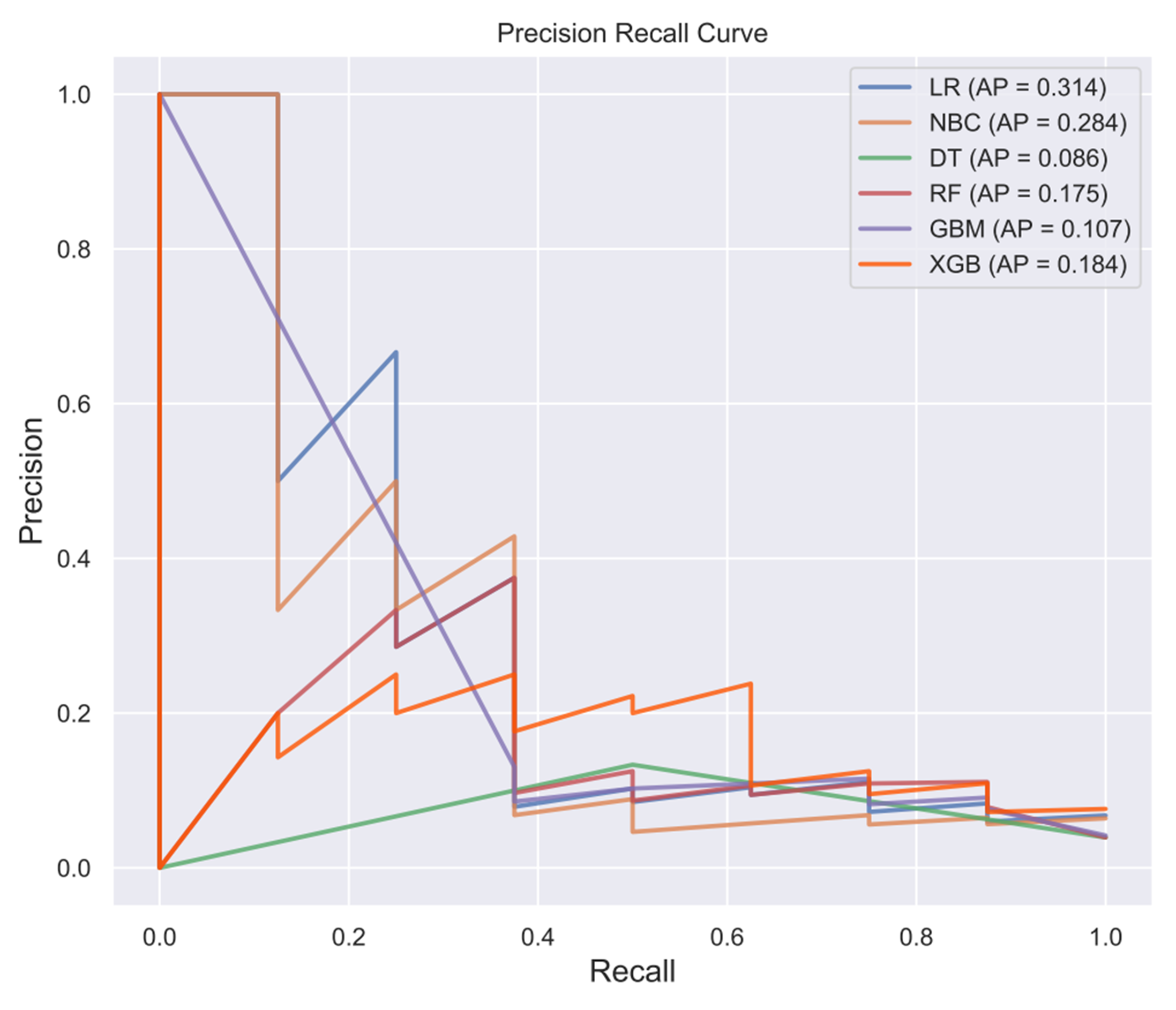

Supplement: Supplementary Figure 1 — Precision recall curve (PRC) for machine learning algorithm. [file Image_1.TIF]
